# Comparable Enhanced Prothrombogenesis in Simple Central Obesity and Metabolic Syndrome

**DOI:** 10.1155/2018/8508549

**Published:** 2018-03-26

**Authors:** Noor Shafina Mohd Nor, Hanis Saimin, Thuhairah Rahman, Suraya Abdul Razak, Nadzimah Mohd Nasir, Zaliha Ismail, Hapizah Mohd Nawawi

**Affiliations:** ^1^Institute for Pathology, Laboratory and Forensic Medicine (I-PPerForM), Universiti Teknologi MARA (UiTM), 47000 Sungai Buloh, Selangor, Malaysia; ^2^Faculty of Medicine, Universiti Teknologi MARA (UiTM), 47000 Sungai Buloh, Selangor, Malaysia

## Abstract

**Objective:**

There is limited data comparing prothrombogenic or fibrinolysis biomarkers (tissue plasminogen activator (tPA) and plasminogen activator inhibitor-1 (PAI-1)) simultaneously in subjects with Metabolic Syndrome (MS), simple central obesity without MS (COB) and normal controls (NC). We investigated the concentrations of fibrinolysis biomarkers in subjects with MS, COB and NC.

**Methods:**

A cross-sectional study involving 503 drug naive subjects (163 males, aged 30–65 years old (mean age ± SD = 47.4 ± 8.3 years)) divided into MS, COB and NC groups. COB was defined as central obesity (waist circumference (WC) males ≥90 cm, females ≥80 cm) in the absence of MS according to the International Diabetes Federation 2006. Fasting blood levels of tPA and PAI-1were analyzed.

**Results:**

MS and COB had significantly higher concentration of all biomarkers compared to NC. The MS group had significantly higher concentration of tPA and PAI-1 compared to COB. WC and HDL-c had significant correlation with all biomarkers (tPA *p* < 0.001, PAI-1 *p* < 0.001). Fasting plasma glucose and diastolic blood pressure were independent predictors after correcting for confounding factors.

**Conclusion:**

Central obesity with or without MS both demonstrated enhanced prothrombogenesis. This suggests that simple obesity possibly increases the risk of coronary artery disease in part, via increased susceptibility to thrombogenesis.

## 1. Introduction

Metabolic Syndrome (MS) is currently recognized as a major public health problem [1]. It represents a cluster of risk factors consisting of central obesity, atherogenic dyslipidemia, hypertension, hyperglycemia and insulin resistance. Based on the data from the National Health and Nutrition Examination Survey, the age-adjusted prevalence of metabolic syndrome was 38.5% for all subjects, 41.9% for men, and 35.0% for women [2]. It is estimated that approximately 20–25% of the South Asian population has MS [3]. MS has also been well reported to augment the risk of type 2 diabetes mellitus (T2DM) and cardiovascular diseases [4–6].

Previous data have reported that MS, obesity and hyperinsulinemia are associated with increased inflammation and endothelial dysfunction [7–10] which are partly responsible for the development of atherosclerosis and complication of diabetes. Furthermore, MS and obesity have also been linked to higher concentrations of prothrombogenic or fibrinolysis biomarkers for instance tissue plasminogen activator (tPA) and plasminogen activator inhibitor-1 (PAI-1). MS has features of a hypercoagulable state involving higher levels of clotting factors and inhibition of the fibrinolytic pathway [11].

This fibrinolytic activity is dependent on the balance between tPA and PAI-1 [12]. Impaired function of fibrinolytic system is expected to lead to progression of vascular disease and predispose to higher risk of vascular thrombosis. Higher PAI-1 level was documented in MS subjects, which suggested that the tilt toward a more prothrombotic and proinflammatory milieu in the vascular endothelium may be pathognomonic of MS [13]. On the other hand, Michalska et al. reported higher concentration of tPA and PAI-1 in subjects with morbid obesity compared to normal control [14].

However, there is very limited data to date comparing all these fibrinolysis biomarkers simultaneously in subjects with MS, simple central obesity without Metabolic Syndrome (COB) and normal controls (NC). Therefore, the aims of this present study were to: (1) compare the concentrations of fibrinolysis biomarkers between subjects with MS, COB and NC; (2) assess the association between obesity categories of subjects with these markers; (3) evaluate the correlation of the fibrinolysis biomarkers with various MS parameters and (4) determine whether any of the MS parameters are independent predictors for these markers after correcting for confounding factors.

## 2. Materials and Methods

A cross-sectional study was conducted in Specialist Clinics of our Institution and also the community health screening programs. All patients gave their written informed consent and approval from the Institutional Research Ethics Committee (reference code: 600-RMI (5/1/6/01)) was obtained before commencement of the study. Based on PAI-1 from a study by Ahirwar et al., 95% CI and power of 80%, using PS software for comparing mean with the ratio of (MS:COB:NC) as 1:1:1 the minimum sample size required for each group was 60^13^. A total of 503 participants completed a set of questionnaire and a full history exploring smoking habits, alcohol consumption and family history of premature coronary heart disease (CHD) were also obtained from them. Anthropometric, blood pressure measurement, and selected biochemicals analysis were collected from their records.

### 2.1. Anthropometric and Blood Pressure Measurement

Anthropometric measurements including weight, height, body mass index (BMI) and WC were obtained using standardized methods by trained professionals. Weight and height were measured to the nearest 0.1 kg and 0.01 m respectively in light clothing without shoes using a pre-calibrated SECA digital scale and height rod. BMI (kg/m^2^) was calculated using the formula: weight (kg) divided by squared height (m^2^), while WC was measured using a measuring tape midway between the inferior margin of the rib and the topmost palpable border of iliac crest (to the nearest 0.5 cm). An automated BP monitor (Omron, USA) was used to measure BP, applied on the right arm held at heart level with the participants in seated position following at least 5-minute rest. Three measurements were taken and the average of the last two readings were taken as the participant's BP.

### 2.2. Biochemical Analysis

Subjects were required to fast for at least 8 hours prior to blood taking. Fasting venous blood samples were collected and centrifuged (at 1,645 g for 7 minutes) as soon as possible or within an acceptable delayed timeframe of between 30 and 45 minutes. Plasma and serum were aliquoted and stored at −20°C until analysis. The enzymatic reference method with hexokinase, standardized against Isotope Dilution Mass Spectrometry was used for FPG assay while total cholesterol (TC), TG and HDL-c were assayed by enzymatic reference methods on an automated analyzer (Cobas Integra 400 plus, Roche Systems, Germany). Low density lipoprotein cholesterol (LDL-c) was calculated using Friedewald equation). The intra- and inter-assay coefficient of variation (CV) for FPG, TC, TG and HDL-c were 1.8% and 2.1%, 0.5% and 1.9%, 1.6% and 1.9% and 1.1% and 1.0%; respectively.

### 2.3. Fibrinolysis Biomarkers Analyses

Fibrinolysis biomarkers measured were tPA and PAI-1. They were analyzed by using sandwich ELISA methods.

### 2.4. Inclusion and Exclusion Criteria

The 2006 International Diabetes Federation (IDF) for Asians definition was used to define MS. MS is considered in participants with central obesity, defined as waist circumference (WC) for males ≥ 90 cm and females ≥ 80 cm with evidence of at least 2 out of 4 of the following criteria: elevated fasting plasma glucose (FPG) ≥ 5.6 mmol/L, elevated BP (systolic BP (SBP) ≥ 130 mmHg and/or diastolic BP (DBP) ≥ 85 mmHg), elevated triglycerides (TG) ≥ 1.7 mmol/L or reduced high density lipoprotein cholesterol (HDL-c) < 1.0 mmol/L in men and <1.3 mmol/L in women.

COB group on the other hand, was considered for subjects with central obesity based on the waist circumference plus 0 to 1 other criteria for MS and NC were those with WC < 90 cm for males and < 80 cm for women, with no other criteria for MS (FPG < 5.6 mmol/L, BP < 130/85 mmHg, TG ≤ 1.7 mmol/L and HDL-c ≥ 1.0 mmol/L for men and > 1.3 mmol/L in women).

Subjects who were on oral hypoglycemic agents (OHA), insulin, anti-hypertensive agents, lipid lowering agents, on long-term antioxidant or anti-inflammatory therapy and those with chronic inflammatory disorders, malignancy or severe diseases that shorten life expectancy were excluded from this study. The subjects with DM, hypertension and/or hyperlipidemia were recruited only if they were either newly diagnosed and/or drug naive in terms of anti-diabetic, anti-hypertensive and lipid lowering medications.

### 2.5. Statistical Analysis

Statistical analyses were performed using Statistical Program for Social Science Software (SPSS) (Version 23, Chicago, IL). Data were presented as mean ± standard deviation (SD) or percentage (%) and the significance was set at *p* < 0.05. Differences among groups were analyzed using one-way ANOVA, with the Bonferroni post-hoc group wise comparisons. For parametric statistics, Pearson's correlation coefficient test was employed to examine the associations. Association between categorical data was assessed using Chi-squared test. Simple linear logistic regression analysis was used to identify the independent predictors for fibrinolysis biomarkers.

## 3. Results

A total of 503 subjects aged 30 to 65 years old (mean age ± SD = 47.4 ± 8.3 years) were divided into the three groups; Metabolic Syndrome (MS), simple central obesity without MS (COB) and normal controls (NC). A total of 223 participants were group into MS group, 182 into COB group and 98 into the NC group. The three groups were matched for age, gender, ethnicity and smoking status (*p* > 0.05).

The baseline and laboratory characteristics of the 503 participants according to group (MS, COB and NC) are depicted in Table 1. The MS group showed significantly higher WC, BMI, systolic and diastolic BP compared to COB and NC (*p* < 0.001). Furthermore, the MS group also had significantly higher LDL-c (<0.01), TG (*p* < 0.001) and TC (*p* < 0.001), but significantly lower HDL-c (*p* < 0.001) compared to COB and NC.

As presented in Table 2, analysis of all subjects demonstrated that both the MS and COB groups had significantly higher concentration of all biomarkers compared to NC. The MS group had significantly higher concentration of tPA and PAI-1 compared to COB.

Following separate analysis with respect to different gender, both males and females showed that PAI-1 concentration was not significantly different between COB versus NC and MS versus COB. With regard to tPA, only males showed no significant difference between MS versus COB group (Table 2). Significantly higher proportions of the MS subjects were found in the highest quartile for tPA and PAI-1 compared to COB and NC (*p* < 0.001 and *p* < 0.01 resp.) (Table 3).

This study showed that WC and HDL-c had significant correlation with both biomarkers (*p* < 0.001 for both). The other significant correlations are also depicted in Table 4.

With regards to the independent predictors for the biomarkers, our study showed that WC, FPG and DBP were the independent predictors for tPA (*p*=0.004, *p* < 0.001, *p* < 0.01 resp.) and FPG and DBP for PAI-1 (*p*=0.005, *p*=0.042 resp.) after correcting for confounding factors (age, gender, ethnicity, smoking status and lipid profile). Separate gender analyses revealed that for females, WC, FPG and DBP were the independent predictors for tPA (*p*=0.001,   < 0.001, *p*=0.035 resp.) and HDL-c for PAI-1 (*p*=0.042); whereas in males TG and FPG were the independent predictors for tPA (*p*=0.04, *p* < 0.001 resp.) (Table 5).

## 4. Discussion

Our study evaluated the fibrinolysis or prothrombogenic biomarkers simultaneously in MS, simple central obesity without the presence of MS and normal controls. To the best of our knowledge, there is very limited study addressing this matter simultaneously in these three different groups to date. Our results showed that the MS group had significantly higher levels of all biomarkers compared to normal controls. This is in agreement with another previous published study by Ahirwar et al. However, their subjects were much smaller in size compared to our study and compared only between MS and normal controls [13].

The COB group also showed an increased prothrombotic state compared to normal controls evidenced by significantly elevated concentration of all biomarkers. Previous studies have also reported higher fibrinolysis biomarkers in obese subjects [14–16]. However, these studies did not specify the MS status among the subjects in the obesity group as in this present study which will allow better understanding of the effect of the metabolic profile on the corresponding biomarkers concentration.

This study further highlights the strong correlation between the MS components (central obesity, hypertension, plasma glucose and cholesterol) and these biomarkers which explains the higher concentrations of these biomarkers among MS compared to COB subjects. Such observations emphasizes the need to optimize management of these MS components in order to reduce prothrombogenesis among MS subjects which could subsequently reduce the risk of coronary artery disease.

In addition, although there are several reports of prothrombogenic status in MS cohorts, very scarce data is currently available in COB in the absence of MS. Our study clearly illustrated that both categories of central obesity that is MS and COB even in the absence of MS had higher tPA and PAI-1 concentrations compared to NC with MS group having significantly higher concentration compared to COB group. This suggests a dose-dependent effect and therefore, a potential benefit provided by these biomarkers in identifying the risk of progression into further complications.

However, segregation according to gender revealed that both genders had comparable prothrombogenesis between the MS and NC. However, among males, it appears that there are no significant differences when compared between COB and NC as well as COB and MS, suggesting the possibility of different tissue adiposity or insulin sensitibity between the two genders which could influence the biomarkers [17]. Therefore, our findings suggest that each biomarker should be interpreted accordingly, with different emphasis in males and females.

Furthermore, this study also suggests that central obesity alone irrespective of metabolic profile may act as an important contributor to enhanced prothrombogenesis. One study by Gómez-Ambrosi et al. which divided the subjects into lean, metabolically healthy obesity (MHO) and metabolically abnormal obesity (MAO), reported that the prothrombogenic biomarker concentrations were significantly increased in both obese groups with no differences between them [18]. However, contrary to ours this study looked at different biomarkers (fibrinogen and homocysteine) and did not examine the tPA and PAI-1 concentrations and included patients who were already on antihypertensive and/or lipid-lowering medications. Hence, the novelty of this present study is the recruitment of drug naïve patients that would reduce the possible confounding factors which may interfere with the interpretation of findings.

Our present study, which is the first large cross-sectional study amongst predominantly Malay Asian population clearly revealed that significantly higher number of MS subjects belonged to the higher quartile categories of fibrinolysis biomarkers compared to COB and NC. This highlights the association of the biomarkers with obesity categories and suggests that enhanced prothrombogenesis is seen more commonly with worsening obesity and clinical condition spectrum.

WC and c-HDL were demonstrated in our study to be the two parameters which significantly correlated with all biomarkers of prothrombogenesis. Our findings are parallel to those reported by Aziz et al. [19] who showed that WC was significantly correlated with tPA and PAI-1. However, this study did not examine the other parameters of MS and moreover had smaller sample size (*n*=28) compared to ours.

In this current study, we demonstrated that FPG and DBP were the independent predictors for both tPA and PAI-1 after adjusting for age, gender, ethnicity, smoking status and lipid profile. Apart from that, WC was another independent predictor for tPA. Following separate analyses according to different genders, in females, WC, FPG and DBP were still the independent predictors for tPA. However, in males only FPG was noted as independent predictor for tPA with the addition of TG. In relation to PAI-1, only HDL was identified as the independent predictor in females. No independent predictor was noted for PAI-1 in males. This again suggests that each fibrinolysis biomarkers is influenced in different ways by the MS parameters. Therefore, the planning of future intervention needs to take into account gender difference to ensure better outcome. Schoenhard et al. previously reported that BMI, systolic and diastolic blood pressure, total cholesterol, glucose, and triglycerides were all significant predictors of t-PA and PAI-1 in both females and males, however, the study was for a very different study population compared to ours [20].

An important strength of the present study is the involvement of a large number of participants who were not exposed to any lifestyle or therapeutic intervention that is drug naïve where they were not on anti-diabetic, anti-hypertensive, lipid-lowering agents and/or long-term antioxidant or anti-inflammatory therapy which may act as a confounding factor. However, a perceived limitation is that the study is cross-sectional and hence is only able to show association but not causal effect. Future studies focusing on longitudinal data are seen crucial to provide extended knowledge on patients along different spectrum of clinical disease severity as they progresses from normal weight to simple central obesity and MS.

## 5. Conclusion

In conclusion, central obesity with or even without MS both demonstrated elevated prothrobogenic or fibrinolysis biomarkers in drug naïve predominantly Malay Asian population. This suggests that central obesity possibly increases the risk of coronary artery disease in part, via increased susceptibility to thrombogenesis. WC and HDL-c are persistently significantly correlated with all biomarkers of prothrombogenesis. Hence, the fibrinolysis biomarkers are potentially ideal markers to guide the appropriate therapeutic interventions in centrally obese patients even in the absence of MS, to reduce progression into the development of complications such as cardiovascular diseases and T2DM.

## Figures and Tables

**Table 1:** Baseline characteristics of subjects according to groups.

Parameters	MS (*n*=223)	COB (*n*=182)	NC (*n*=98)	*p* value^1^ (MS versus NC)	*p* value^2^ (COB versus NC)	*p* value^3^ (MS versus COB)
Age (years)	48.7 ± 8.4	47.0 ± 8.5	46.2 ± 7.8	NS	NS	NS
^b^Gender (% male/Female)	(36.8/63.2)	(28.0/72.0)	(34.4/65.6)	NS	NS	NS
^b^Ethnicity (% Malay/Chinese/Indian/Bumiputera)	96.9/0.9/1.3/0.9	96.7/0.5/2.7/0.0	96.9/1.0/0.0/2.1	NS	NS	NS
^b^Current smoker (%)	12.1	8.8	14.3	NS	NS	NS
^a^BMI (kg/m^2^)	29.5 ± 4.1	28.9 ± 4.3	21.9 ± 2.5	^∗∗∗^	^∗∗∗^	NS
^a^WC (cm)	94.4 ± 8.8	93.0 ± 9.7	73.8 ± 7.7	^∗∗∗^	^∗∗∗^	NS
^a^Systolic BP (mmHg)	138.7 ± 22.4	125.4 ± 16.5	112.8 ± 10.0	^∗∗∗^	^∗∗∗^	^∗∗∗^
^a^Diastolic BP (mmHg)	84.5 ± 12.8	78.4 ± 10.4	69.9 ± 8.4	^∗∗∗^	^∗∗∗^	^∗∗∗^
^a^TC (mmol/L)	6.0 ± 1.1	5.6 ± 0.8	5.6 ± 1.0	^∗^	NS	^∗∗∗^
^a^TG (mmol/L)	2.3 ± 1.2	1.3 ± 0.4	1.0 ± 0.3	^∗∗∗^	^∗∗∗^	^∗∗∗^
^a^HDL-c (mmol/L)	1.1 ± 0.3	1.4 ± 0.3	1.6 ± 0.4	^∗∗∗^	^∗∗∗^	^∗∗∗^
^a^LDL-c (mmol/L)	3.8 ± 1.0	3.6 ± 0.7	3.5 ± 1.0	NS	NS	^∗^
^a^FPG (mmol/L)	7.4 ± 3.3	5.1 ± 0.6	4.9 ± 0.5	^∗∗∗^	^∗^	^∗∗∗^

Data are expressed as ^a^mean ± SD, or ^b^ percentage. ^∗^*p* < 0.05, ^∗∗^*p* < 0.01, ^∗∗∗^*p* < 0.001; NS: not significant; MS: metabolic syndrome; COB: simple central obesity; NC: normal controls; BMI: body mass index; WC: waist circumference; BP: blood pressure; TC: total cholesterol; TG: triglycerides; HDL-c: high density lipoprotein cholesterol; LDL-c: low density lipoprotein cholesterol; FPG: fasting plasma glucose.

**Table 2:** Comparison of fibrinolysis biomarkers concentration between groups in all subjects and according to gender.

Biomarker	MS (*n*=223)	COB (*n*=182)	NC (*n*=98)	*p* value^1^ (MS versus NC)	*p* value^2^ (COB versus NC)	*p* value^3^ (MS versus COB)
*All subjects*						
tPA (ng/ml)	6.12 ± 5.97	4.40 ± 4.39	2.92 ± 2.20	^∗∗∗^	^∗∗^	^∗∗^
PAI-1 (ng/ml)	70.95 ± 59.85	57.82 ± 49.22	43.74 ± 30.65	^∗∗∗^	^∗^	^∗^

*Males*						
tPA (ng/ml)	6.38 ± 6.10	4.78 ± 4.31	2.94 ± 1.90	^∗∗^	^∗^	NS
PAI-1 (ng/ml)	83.61 ± 76.04	62.65 ± 43.28	45.37 ± 33.76	^∗∗^	NS	NS

*Females*						
tPA (ng/ml)	5.97 ± 5.92	4.18 ± 4.43	2.90 ± 2.33	^∗∗∗^	^∗^	^∗∗^
PAI-1 (ng/ml)	64.07 ± 47.79	55.95 ± 51.40	42.74 ± 28.87	^∗∗^	NS	NS

Data are expressed as mean ± SD. ^∗^*p* < 0.05, ^∗∗^*p* < 0.01, ^∗∗∗^*p* < 0.001; NS: not significant; MS: metabolic syndrome; COB: simple central obesity; NC: normal controls; tPA: tissue plasminogen activator; PAI-1: plasminogen activator inhibitor.

**Table 3:** Association between obesity categories of subjects with quartiles of fibrinolysis biomarkers.

Biomarkers	MS *n* (%)	COB *n* (%)	NC *n* (%)	*p* value
*tPA (ng/ml)*				
Q1: <2.66	53 (24.9)	77 (42.3)	52 (53.6)	**<0.001**
Q2: 2.66–4.41	54 (25.4)	50 (27.5)	29 (29.9)	
Q3: 4.42–7.19	54 (25.4)	33 (18.1)	12 (12.4)	
Q4: > 7.19	52 (24.4)	22 (12.1)	4 (4.1)	

*PAI-1 (ng/ml)*				
Q1: <27.80	41 (20.8)	38 (25.2)	33 (39.3)	**<0.01**
Q2: 27.80–45.09	52 (26.4)	40 (26.5)	20 (23.8)	
Q3: 45.10–76.83	50 (25.4)	42 (27.8)	20 (23.8)	
Q4: >76.83	54 (27.4)	31 (20.5)	11 (13.1)	

MS: metabolic syndrome; COB: simple central obesity; NC: normal controls; tPA: tissue plasminogen activator; PAI-1: plasminogen activator inhibitor.

**Table 4:** Correlation of fibrinolysis biomarkers with MS parameters.

Biomarkers	MS parameters	Pearson correlation	*p* value
tPA (ng/ml)	WC	0.167	*p* < 0.001
	TG	0.160	*p* < 0.001
	HDL-c	−0.175	*p* < 0.001
	FPG	0.151	*p* < 0.001

PAI-1 (ng/ml)	WC	0.171	*p* < 0.001
	HDL-c	−0.155	*p* < 0.001
	SBP	0.129	*p* < 0.01
	DBP	0.175	*p* < 0.001

tPA: tissue plasminogen activator; PAI-1: plasminogen activator inhibitor; WC: waist circumference; SBP: systolic blood pressure; DBP: diastolic blood pressure; TG: triglycerides; HDL-c: high density lipoprotein cholesterol; FPG: fasting plasma glucose.

**Table 5:** Independent predictors for the fibrinolysis biomarkers.

Variables	Independent predictors	Constant	Beta	SE	OR	95% CI	*p* value
*All subjects*							
tPA	WC	1.703	0.147	0.021	0.061	0.020, 0.103	0.004
	FPG		−0.172	0.478	2.993	2.054, 3.933	<0.001
	DBP		0.148	0.027	−0.069	−0.122, −0.017	0.01
PAI-1	FPG	5.514	0.133	5.585	15.723	4.746, 26.701	0.005
	DBP		0.148	0.303	0.619	0.024, 1.214	0.042

*Females*							
tPA	WC	−0.849	0.193	0.025	0.081	0.032, 0.130	0.001
	FPG		0.283	0.558	2.978	1.879, 4.076	<0.001
	DBP		−0.164	0.030	−0.064	−0.124, −0.005	0.035
PAI-1	HDL-c	46.557	−0.143	9.159	−18.671	−36.697, −0.644	0.042

*Males*							
tPA	TG	7.571	0.183	0.451	0.917	0.025, 1.809	0.04
	FPG		0.272	0.933	3.297	1.454, 5.141	<0.001

The model reasonably fits well. Model assumptions are met. There are no interaction and multicollinearity problem. tPA: tissue plasminogen activator; PAI-1: plasminogen activator inhibitor; WC: waist circumference; FPG: fasting plasma glucose; SBP: systolic blood pressure; DBP: diastolic blood pressure; HDL-c: high density lipoprotein cholesterol.
